# Cell Migration Assays and Their Application to Wound Healing Assays—A Critical Review

**DOI:** 10.3390/mi15060720

**Published:** 2024-05-29

**Authors:** Chun Yang, Di Yin, Hongbo Zhang, Ildiko Badea, Shih-Mo Yang, Wenjun Zhang

**Affiliations:** 1 School of Mechanical Engineering, Donghua University, Shanghai 200051, China; chy416@mail.usask.ca; 2 Division of Biomedical Engineering, University of Saskatchewan, Saskatoon, SK S7N 5A9, Canada; 3 School of Mechanical and Power Engineering, East China University of Science and Technology, Shanghai 200237, China; zhizhuxiaoscar@gmail.com (D.Y.); hbzhang@ecust.edu.cn (H.Z.); 4 College of Pharmacy and Nutrition, University of Saskatchewan, Saskatoon, SK S7N 5A9, Canada; ildiko.badea@usask.ca; 5 School of Mechatronic Engineering and Automation, Shanghai University, Shanghai 200444, China

**Keywords:** cell migration assay, wound healing assay, system and design perspective

## Abstract

In recent years, cell migration assays (CMAs) have emerged as a tool to study the migration of cells along with their physiological responses under various stimuli, including both mechanical and bio-chemical properties. CMAs are a generic system in that they support various biological applications, such as wound healing assays. In this paper, we review the development of the CMA in the context of its application to wound healing assays. As such, the wound healing assay will be used to derive the requirements on CMAs. This paper will provide a comprehensive and critical review of the development of CMAs along with their application to wound healing assays. One salient feature of our methodology in this paper is the application of the so-called design thinking; namely we define the requirements of CMAs first and then take them as a benchmark for various developments of CMAs in the literature. The state-of-the-art CMAs are compared with this benchmark to derive the knowledge and technological gap with CMAs in the literature. We will also discuss future research directions for the CMA together with its application to wound healing assays.

## 1. Introduction

In the past decade, organ-on-chips technologies have emerged as an important technology for ex vivo studies in biomedical and pharmaceutical applications [1,2,3,4]. Among them, cell migration assays (CMAs), which are fundamental, provide a generic engineered environment for cells and tissues, where cell movements (also called cell migrations) can be examined quantitatively. In the following sections, the term ‘assay’ and the term ‘device’ are used interchangeably. The main idea of enabling cells to migrate in the CMA is to create a cell-free zone (CFZ), and thus cells can slowly move into the CFZ under stimuli [5]. For the convenience of later discussions, such a CMA may be called a “CFZ-based” CMA, and it is cautious about other ways to enable cells to move.

In recent years, CMAs have been applied to the study of cell wound healing, leading to a special kind of CMA for wound healing called wound healing assays, which are used for drug development and a basic understanding of wound healing mechanisms [6,7,8]. The general processes on wound healing assays include wounding and healing. Wounding or making cells wounded is based on the CFZ-based CMA; particularly, the wounded area is a CFZ, where there is no cell (theoretically). Healing is thus about how cells surrounding the CFZ move into the CFZ and occupy it. Naturally, the information on the (1) fullness of the occupation of the cells in the CFZ and (2) the time to reach a certain degree of fullness will be used to measure the healing process. Further, one can study the effects of various stimuli (including drug and nutrition, force, heat, light, etc.).

There are two approaches in the literature to creating CFZs: (a) cell depletion and (b) cell exclusion [9]. In the cell depletion approach, cells are first seeded in CFZs, and then these cells are wounded [10,11]. In the cell exclusion approach, the growth of cells into the CFZs is hindered using physical barriers [12,13,14,15,16]. The cell depletion approach causes considerable damage to cells at the edge of CFZs, while the cell exclusion approach causes minimum damage. However, the cell exclusion approach makes less sense for cell wounding. Another problem with the cell exclusion approach is that the presence of physical barriers could significantly compromise the consistency of CFZs due to significant variations of cell adhesion to the barriers [17]. This paper concentrates on the cell depletion approach.

This paper provides a review of the development of CMAs with an overall goal to identify the knowledge and technology gap, i.e., the discrepancy between what is required and what is achieved on the CMA with its application to the wound-healing process. The paper is organized as follows. In Section 2, existing CMAs in the literature as well as those commercially available along with their classification are presented. In Section 3, we take a design perspective to the CMA to derive its knowledge and technology gap. In Section 4, we discuss other factors relevant to wound healing assays, which helps to give a complete understanding of the limitation of CMAs. Finally (in Section 5), we discuss the future direction of research on CMAs together with the wound healing assay.

## 2. Classification of CMAs

In this paper, we view the classification of a device, the CMA in this case, as the design of the device to meet the design requirement. This approach allows us to derive knowledge and technology gaps by comparing the state-of-the-art device with the design requirement. It is to be noted that the basic information regarding design is documented in the Supplementary Materials along with this paper [18,19,20,21,22,23].

From a point of view of system design, the overall function requirement (FR) of the CMA is expressed as an input–output relation [24,25,26], where the input includes cells with a certain state and property, and the output includes the movement of the cells, i.e., the physiological response of the cells to various stimuli or excitations to the cells during the movement of cells within the CMA. It is to be noted that both stimuli and excitation are also created by the CMA. Further, if a system is decomposed into sub-systems, the classification is applicable to each sub-system, respectively, which means that the overall function of the system may be further divided into a set of sub-functions. In the case of CMAs, the overall FR can be further decomposed into three sub-FRs including: FR1 (to create CFZs), FR2 (to measure the migration of cells), and FR3 (to create the mechanical, chemical, and biological conditions). In the following sections, the classification of the CMAs is presented pertinent to the three sub-FRs.

### 2.1. Creation of CFZs (FR1)

There are two approaches to creating CFZs (FR1), namely cell depletion and cell exclusion, and this paper only discusses the cell depletion approach, see the previous discussion. Cell depletion (DP1) can be classified into five groups in terms of the principles of cell depletion: mechanical (DP1-1), chemical (DP1-2), electrical (DP1-3), thermal (DP1-4), and optical (DP1-5), see Table 1. It is noted that using the abbreviations such as FR and DP (i.e., design parameter) in this paper is to make discussions more structurally sound, and this style of writing of the review paper is taken throughout this paper. Details of each principle are discussed below.

#### 2.1.1. Mechanical Depletion Approach

**Figure 1:**
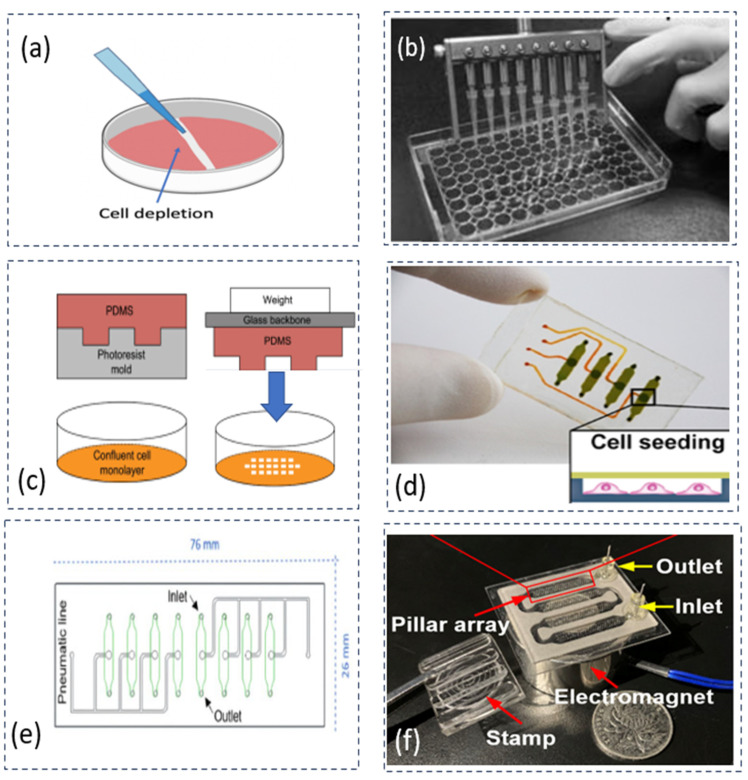
Various CMAs based on the mechanical depletion approach: (**a**) a pipette removing cells manually on a dish plate of cell culture [10], reproduced with the permission of [37]; (**b**) multiple pipettes creating eight CFZs simultaneously based on a commercially available CMA device called ${\mathrm{Oris}}^{\mathrm{TM}}$, adapted with the permission of [28]; (**c**) stamping devices applying consistent forces on cells to improve consistency in the size of CFZs, adapted with the permission of [27]; (**d**) a microfluidic device creating CFZs automatically with the pneumatic actuation by deflecting a flexible membrane, adapted with the permission of [29]; (**e**) a microfluidic device achieving a high consistency of CFZs with various forces, adapted with the permission of [30]; and (**f**) a microfluidic device creating 400 CFZs at one operation in a shear stress condition, adapted with the permission of [31].

Mechanical depletion (DP1-1) removes cells physically by scratching or applying pressure on cells in a defined area. This approach has the advantage of representing mechanical wounding, and it also has the merit of simplicity. The disadvantages of this approach include the poor consistency of CFZs and the difficulty of cleaning the cell debris. Further, the classification of CMA devices in mechanical depletion can be categorized based on the methods of operation: (1) the manual method and (2) automation method. The automation method is a key to improving consistency. The automation method can be further classified into three categories in terms of actuation principles: pneumatic, electromagnetic, and magnetic actuation. Figure 1 shows several devices that are based on the mechanical depletion (DP1-1) approach.

Liang et al. used the mechanical depletion approach to create a CFZ manually by using a pipette (Figure 1a) [10]. The limitation of the manual operation is poor consistency of creating CFZs. To increase the number of samples, Yue et al. developed a device based on the commercially available ${\mathrm{Oris}}^{\mathrm{TM}}$ CMA to create eight CFZs at the same time (see Figure 1b) [28]. The consistency of the CFZs was reported at 6%, with an average wound size of 600 μm × 7 mm. This result was achieved by creating eight CFZs simultaneously using multiple pipette tips. It is noted that the consistency (see Table 1) reported in the current literature is calculated by the relative standard deviation (RSD), as shown in the following equation [30].

(1) $$\mathrm{RSD}\;\left(\%\right)=\frac{\mathrm{SD}}{X}\times 100$$

From the above equation, the standard deviation is denoted as SD, and the average size of CFZs is denoted as X.

To reduce the variation in size of the CFZs, Lee et al. developed a weight stamping device to use a synthetic polymer, Polydimethylsiloxane (PDMS), as a puncher to apply pressure on a layer of cells (see Figure 1c) [27]. The fabrication of such a microfluidic device has made it possible to integrate the channel with sensors for the analysis of cell migration. However, their approach may create a large variation in CFZs because of manual operation. Overall, it is difficult for CMAs with the method of manual operation to meet the requirement of high consistency (about 5% RSD).

To achieve a high consistency of CFZs, Sticker et al. developed a CMA on the microfluidic device capable of creating four samples of CFZs simultaneously with a high consistency (4% RSD) (see Figure 1d) [29]. The CFZs were created by the deflection of the membrane. However, the device had the problem of leaking bubbles at high fluid pressures. Monfared et al. in the same research group, further studied the problem of reproducibility and revealed the limitation in matching the range of shear stresses in vivo [30]. The high pressure of up to 5 bars may result in bubble formation because of the permeability of PDMS. As shown in Figure 1e, the device created four samples of CFZs (5% RSD) with an average area of 1.51 mm^2^ at 76 mm × 26 mm. Yin et al. developed a microfluid device, capable of creating 400 CFZs during one operation at shear stress conditions, as shown in Figure 1f [31]. However, the device has a problem of uniformity (66% in the shear stress condition, indicating high variation at the CFZs). It is difficult to achieve adjustability and uniformity because adjustability inherently produces a source of variations (i.e., non-uniformity).

**Figure 2:**
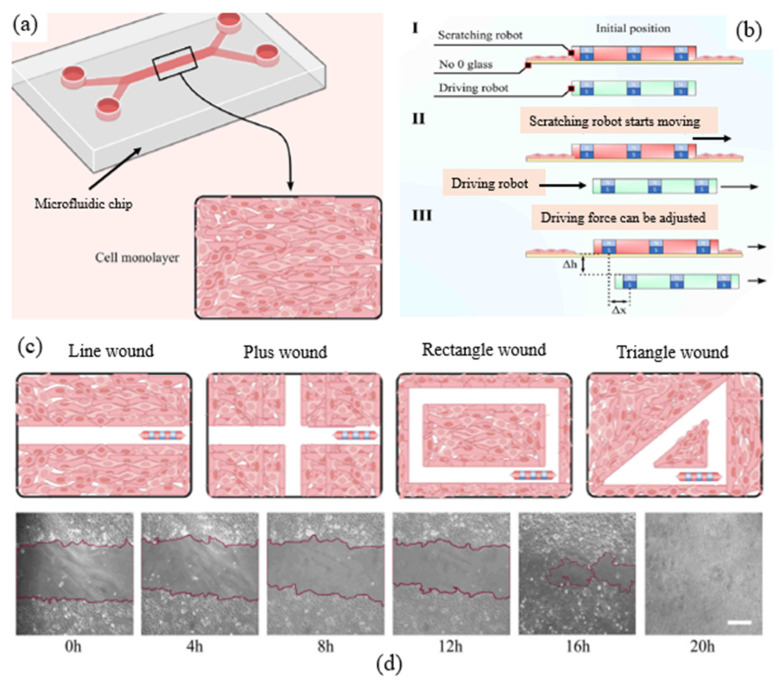
A magnetically controlled robot creating CFZs, adapted with permission from [32]: (**a**) cells cultured on a PDMS-based microfluidic chip; (**b**) the driving robot controls the scratch robot magnetically: (I) initial position of driving robot (green) and scratching robot (red), (II) the scratching robot driven by the driving robot starts moving, (III) the driving force required to move the scratch robot can be adjusted by the distance between the two robots horizontally (∆x) and vertically (∆h); (**c**) schematics of different geometries of CFZs (line wound, plus wound, rectangle wound, and triangle wound); and (**d**) images of line-shaped wounds at different time intervals. Legend white bar is equal to 400 μm.

Yilmaz et al. developed a scratch robot enabling us to create CFZs with different shapes and geometries of CFZs (see in Figure 2a) [32]. The robot is based on magnetic actuation (see in Figure 2b), enabling us to create CFZs of different shapes of wounds (see in Figure 2c) such as line wound, ‘plus’ shape wound, rectangle wound, and triangle wound. According to Yilmaz et al., the consistency of the CFZs can reach 2% [32]. Their study also suggested that wounds with more edges of CFZs may have a faster healing time. However, the device can only create a few samples (i.e., CFZs).

#### 2.1.2. Other Depletion Approaches

The chemical approach (DP1-2) is used to create CFZs through chemical reactions. The advantage of this approach is the possibility to clean the cell debris thoroughly [27]. Lin et al. developed a microfluidic device to create CFZs in fluid shear conditions [33]. However, the disadvantage of this approach is that the use of chemicals (e.g., trypsin) could slow the cell migration process; specifically, the average speed of cell migration in the chemical depletion approach was reduced to 12 μm/h compared to 25 μm/h in the mechanical depletion method [38].

The electrical approach (DP1-3) is used to apply an electrical field to a restricted area among a group of cells [34]. The advantage of this approach is that it increases the ability of automating the wounding process by controlling the electrical signals. However, the major drawback of this approach is that the damage is done to the adjacent cells. Another disadvantage of this approach is the difficulty of cleaning dead cells remaining on the top of electrodes.

The thermal approach (DP1-4) is used to apply heat to a restricted area among a group of cells [35]. The advantage of this approach is it provides the possibility to study healing-induced thermal wounding. The drawback of this approach is that heat may transfer to the surrounding cells, and consequently may negatively affect the viability of cells.

The optical approach (DP1-5) is used to remove cells in a restricted area using the high energy laser ablation [36] and ultraviolet light [39]. This approach has the merits of creating many CFZs, and no direct contact to cells. The disadvantage of this approach is the difficulty of the acquisition of such a specialized instrument. In [36], a commercial laser system called LEAP™ was used to create 96 CFZs simultaneously with a variation at 9% RSD on the CFZs.

### 2.2. Measurement of Cell Migration (FR2)

For the measurement of cell migration (FR2), a common principle is the optical approach (DP2) [17], as mentioned above in this paper. The process of measuring cell migration involves image acquisition and image analysis [40]. For image analysis, deep learning methods [41] have been applied for automated imaging for cell detection and tracking by using positional information based on individual cells [42,43] or a group of cells [44]. However, these studies have limitations in terms of high sensitivity to variations. For image acquisition, there are two options, namely the label-based (DP2-1) method and label-free (DP2-2) method (see Table 2).

For the label-based method (DP2-1), the process of imaging requires attaching chemicals to the cells to detect the change of cell behavior with the chemicals (i.e., known as labels) [45]. The advantage of this method is that the labels absorb visible light to enhance the visual characteristics of cells. However, the labels can be invasive by introducing toxic molecules (e.g., fluorescence) into the cells and affecting the migration. In the study of [46], the resolution of a fluorescence imaging is in the range of 0.4–1 μm. In addition, the device has the limitation that the devices can only measure thin samples (e.g., cell monolayer) with their thickness being about 10 μm.

For the label-free method (DP2-2), the process of imaging does not need to attach chemicals to cells but needs to measure the physical properties (e.g., viscoelasticity) of the response of the group of cells. The advantage of this method is non-invasive, which allows for monitoring cells for days without damaging the cells [45]. Another advantage of devices (e.g., infrared spectroscopes) using this method is that they can measure samples up to a depth of 70 μm [47]. However, this method is limited by relatively poor resolution at the single-cell level (about 5 μm) in imaged-based screening assays [48].

### 2.3. Creation of Mechanical Conditions (FR3)

This paper is focused on the creation of mechanical conditions from the perspective of device development. The function requirement of creating mechanical conditions (FR3) can be decomposed into two sub-functional requirements: (a) to construct a deformation pathway to transmit mechanical energy (FR3a), and (b) to characterize the stress in cells (FR3b). For the construction of a deformation pathway (FR3a), there are three options: fluid-induced pressure (DP3a-1), cell-generated forces (DP3a-2), and environment-induced forces (DP3a-3). For the characterization of stress and strain (FR3b), there are two options: probe-based measurement (DP3b-1) and interaction force measurement (DP3b-2). Table 3 presents the classification of the existing devices to achieve FR3 in terms of the principles of the methods.

#### 2.3.1. Fluid-Induced Pressure

Based on the actuation principle for driving fluids, there are three options: electrical actuation (DP3a-1-1), electromagnetic actuation (DP3a-1-2), and pneumatic actuation (DP3a-1-3). In the following sections, the classification of devices in the existing devices pertinent to the above principles are discussed.

**Figure 3:**
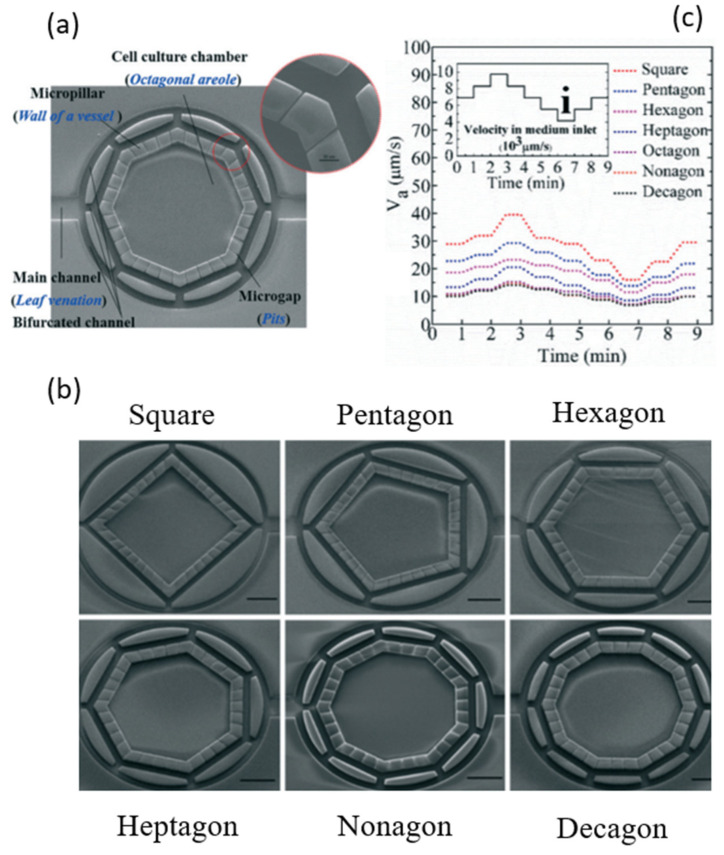
Structural design of a bio-inspired cell chamber for studying the uniformity of flow distribution, adapted with permission from [56]: (**a**) a cell chamber in octagonal shape; (**b**) cell chambers with different shapes including square, pentagon, hexagon, heptagon, nonagon, and decagon; and (**c**) the velocities varying in different shapes of cell chambers with the same inlet velocity. The scale bar is 300 μm.

For the electrical actuation (DP3a-1-1), the existing studies focus on the control of fluid-induced pressure to manipulate shear stress in cells by using pumps or valves in microfluidic devices. Polacheck et al. described microfluidic devices as promising platforms to elicit cell migration in biological systems such as blood vessels [49]. Shemesh et al. reviewed microfluidic devices integrated with analysis tools for providing the shear stress environment for cell migration [50]. Kaarj and Yoon studied devices that deliver mechanical stimuli to cell migration assays [51]. Most of the existing pumps and valves are connected externally, whereas cells are in the microfluidic system [52,53]. Shear stresses generated internally are thus not uniform in microfluidic channels. For example, Saias et al. reported a device with a uniformity of 80% and with a tolerance of 10% [54]. However, such uniformity and tolerance are far from sufficient. Schimek et al. reported on a device where there are bulky external pumps, enabling local stress in cells [55]. Li et al. studied the uniformity of shear stresses in a bio-inspired structure [56], as shown in Figure 3. The limitation with their device was that the uniformity is only in the low range of shear stresses ($10^{-3}$ to $10^{-2}$ dyn/cm^2^), as opposed to a wide range of shear stresses (1–70 dyn/cm^2^) in blood vessels [72,73].

For the electromagnetic actuation (DP3a-1-2), the concept is to use the magnetic field driven by electric power to create the deformation to drive the fluid. It has the potential of controlling the flow precisely. Bhushan et al. proposed a pump system, which achieved a fluid backpressure of 20 kpa with a low energy dissipation of 0.65 mJ per switching and allowed the pump to operate at 0.45 μL/s with an average power dissipation of 1.3 mW and a temperature rise of 0.04 °C [57]. However, the drawback of the electromagnetic actuation is its difficulty in maintaining the temperature at 37 °C due to the high amplitude of the electrical currents.

For the pneumatic actuation (DP3a-1-3), the concept is to use gas or liquid to generate a high mechanical pressure on polymeric diaphragms resulting in a high flow rate at 102.05 μL/min in microfluid channels [58]. The shortcoming of this actuation principle is the formation of bubbles, which may potentially damage cell dynamics.

#### 2.3.2. Cell-Generated Force

**Figure 4:**
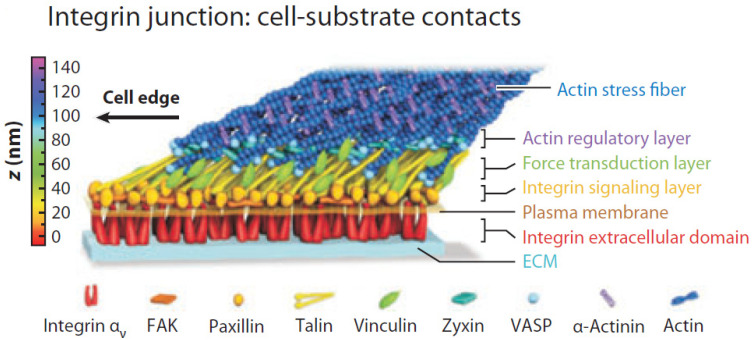
Various signal molecules in the deformation pathway: a protein network (i.e., talin, vinculin, paxillin, focal adhesion kinase (FAK), vasodilator stimulated phosphoprotein (VASP), and other proteins) interconnected between cells and the extracellular matrix (ECM). Adapted with permission from [74].

In the literature, there are two approaches to use cell-generated stresses in cells: the mechanical approach (DP3a-2-1) and the chemical approach (DP3a-2-2). Figure 4 illustrates various signal molecules from 1 to 140 nm in a deformation pathway between a cell and its environment [74]. As shown in Figure 4, the cell environment, often referred to as the extracellular matrix (ECM), is a complex structure serving as the connective tissue to support cells [75].

The mechanical approach (DP3a-2-1) is used to apply external force at the structure of a cell, and the cell then provides the force feedback on the deformation pathway [48]. For any moving object, it can be characterized by stiffness, inertia, and damping. Previous studies gave vague definitions about stiffness compared to “rigidity” [76], “hardness” [77], and “shape stability” [78]. It is noted that, in this paper, stiffness is defined as a system property of a cell with the ability to deform at a particular point of location on the cell along a particular direction (including both translational and rotational) when the cell is subject to a force (including moment) from the environment in which the cell interacts [79,80]. Fu et al. presented micropost arrays to investigate the effect of the stiffness of the substrate on cell shape and cytoskeletal contractility [81]. Nava et al. presented the concept of “anisotropic force” [82], which is generated by the cell on the ECM with different magnitudes of forces at varying orientations [83,84]. Steward and Kelly described the stiffness and cell shape as intrinsic mechanical cues to induce biological responses [85]. Paul et al. presented various simulators to generate stiffness for studying the effects of physical confinement on cell morphology, as well as behavior [86]. Yamada and Sixt defined the stiffness of the cell as a “local” property as opposed to a “bulky” property [87].

The chemical approach (DP3a-2-2) is used to induce the change of mechanical properties (e.g., stiffness) by the chemical cues including ions, signal molecules, and soluble factors [88]. Forero et al. presented that the adhesion forces can last for several tens of seconds at 40–70 pN forces at a protein called fimbriae (7 nm in diameter and 1 μm in length) [89]. The advantage of the chemical approach is that it can accurately target specific proteins. Sun et al. studied the different types of chemical bonds including slip bond, catch bond, and clustering bond for force generation and measurement at the molecular level [90]. However, the disadvantage of the chemical approach is that the structural change may affect cell dynamics, and thus become a confounding factor of the migration of cells.

#### 2.3.3. Environment-Based Force

**Figure 5:**
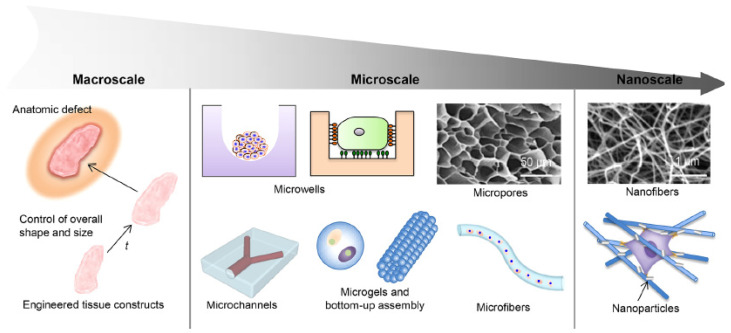
Schematic diagram of the structural design aspects of engineered biomimetic materials in macroscale, microscale, and nanoscale: the design aspects in macroscale are related to external structural characteristics, such as overall shape and size; the design aspects in microscale include microwells, micropores, microchannels, microgels, and microfibers; and the design aspects in nanoscale include nanofibers and nanoparticles. Adapted with permission from [91].

The mechanical forces on cells can be created by various stimuli in the environment. There are two types of stimuli from the environment: physical stimuli (DP3a-3-1) and chemical stimuli (DP3a-3-2). For the physical stimuli method, this method includes optical [61], electric [59,62,64], magnetic [63], and acoustic [65] stimuli. For example, Morimoto et al. used an electric field (2.5 V/mm) to stimulate the contraction of muscle cells, capable of generating mechanical force at 13 mN [59]. The limitation for the physical stimuli method is that it lacks the first principles to generate controllable mechanical forces, making it difficult to control the mechanical forces. Several studies presented synthetic environments (i.e., substrate or matrix) to enhance the deformation at a particular structure on cells, as shown in Figure 5. Huang et al. studied the structures of the synthetic ECM at different scales [91]. Liu et al. presented the development of stimuli-responsive materials (e.g., hydrogels) for studying the mechanical interaction with cells [92].

For the chemical stimuli method, the change of the mechanical properties of the cell environment can be induced by the chemical stimuli by changing the bonds between the cell and its environment. These chemical stimuli include pH [67,70], oxygen [93], and ion concentration [66]. In addition, biomolecules such as enzymes were demonstrated to be effective in changing the stiffness of the cell environment due to the chemical reaction [69,94], crosslink activation [95], and enzymatic degradation [68]. For instance, Yin et al. built a synthetic ECM integrated with hydrogels, enabling a change in the morphology of the ECM with respect to the change of glucose level [69]. The limitation for the chemical stimuli method is that the chemical reaction could take days while the changes of cell morphology occur in minutes or hours [70]. Further, the variation in the chemical properties of the cell environment may not be desirable to the biological environment with living cells.

#### 2.3.4. Characterization of Stress and Strain

There are two general approaches to characterizing stress and strain: probe-based measurement (DP3b-1) and interaction force measurement (DP3b-2) [96,97]. The first approach is used to measure interaction forces between a probe tip and sample surface in a probe-based instrument, such as an atomic force microscope (AFM) [71]. This approach can provide a high resolution (5 pN to 10 nN) but has a high level of intrusiveness, which may cause irreversible damage of cell structures and eventually lead to cell death [98]. The second approach is used to measure the interaction traction force between cells and the synthetic ECM [99,100]. The interaction force instrument, such as traction force microscopy (TFM), is used to measure the adhesion force between cells and the substrate. This approach can provide the resolution between 2 and 120 nN at a 2-dimensional surface [60]. However, the limitation of this approach is the low accuracy in measuring forces due to the disturbance from the dynamic interaction between cells and their environment.

#### 2.3.5. Combination of Different Conditions

Most of the existing CMAs are focused on creating multiple chemical conditions for drug testing in wound healing assays. It is noted that in this paper, the number of conditions means that the number of varieties of geometric distributions of fluids (i.e., drugs or growth factors) and the number of samples means the number of CFZs created simultaneously. Lee et al. developed a “stamping” device based on PDMS with one condition and 20 samples [27]. Yue et al. developed a device to change 16 chemical conditions based on a commercially available CMA (${\mathrm{Oris}}^{\mathrm{TM}}$) enabling us to produce eight samples simultaneously by multichannel pipettes [28]. To combine multiple conditions with multiple samples, Sticker et al. developed microfluidic devices with four conditions and four samples [29]. The fluidic channels were boned with a deformable PDMS membrane with a diameter of 1.5 mm and height of 250 μm. Lin et al. developed a microfluidic device with three shear stress conditions and three samples [33]. Monfared et al. presented a device consisting of five substrates, and there were eight conditions and four samples on each substrate [30]. Yin et al. developed a microfluidic device with one condition and 400 samples [31]. However, none of the existing CMAs can achieve more (about 100) chemical conditions, many samples (greater than 100), and the strain condition.

## 3. Discussion

Based on the above discussion, we shall be able to conclude that the requirement on CMAs can be decomposed into several sub-requirements in terms of processes as follows: (1) achieving the high consistency of CFZs (about 5% RSD) (**R1**), (2) enabling the creation of different shapes of CFZs for the study of healing (**R2**), (3) enabling them to match the magnitude of shear stresses (from 1 to 70 dyn/cm^2^) to the range of shear stresses in vivo (**R3**), (4) achieving the uniformity of the generated shear stresses with 80% relative error in the distribution of shear stresses (**R4**), and (5) enabling them to make many mechanical, chemical, and biological conditions for drug testing (**R5**).

Among all the above five sub-function requirements, sub-functions (1, 3) are the two most important FRs. Sub-functions (2, 4) are constraint requirements (CRs) in that they create the contexts for the device to be more biomimetic. Sub-function 5 is related to the performance requirement (PR) of the device, because there could be a target value to evaluate how good the function is played by the device. For instance, the number of conditions is the target value to evaluate the efficiency for drug development. A comprehensive clarification of the design requirements (i.e., FR, CR, and PR) and function decomposition can be found in the Supplemental Material. In the following sections, knowledge and/or technological gaps in building CMAs in the context of their applications to the wound-healing process will be derived from the requirement on CMAs, as concluded above, and the state-of-the-art CMAs presented in Table 4.

### 3.1. Consistency of CFZs (R1)

There are three drawbacks with the existing CMAs in meeting the requirement R1 (i.e., required consistency): (a) the presence of cell damage at the edge of CFZs, (b) the presence of cell debris in the CFZs, and (c) the high degree of manual operations in the process to create CFZs. Regarding (a) and (b), though the chemical depletion can create “clean” CFZs [33], the disadvantage of the chemical depletion is that it is difficult to achieve accurate shapes of CFZs. In the study of [38], the migration of cells significantly slowed down to 12 μm/h (average 25 μm/h) by using the chemical approach. Mechanical depletion has the merit of simplicity, and it has the advantage of simulating the process of mechanical wounding; however, there is no effective method available to “clean” CFZs after the operation of the mechanical depletion. Regarding (c), the existing “scratch-based” CMA is usually completed with manual operation, which is inherently a source of inconsistency [10]. The existing CMA (based on mechanical depletion) can only create one or few CFZs, and therefore the CMA can only be used to conduct the experiment of cell migration on few samples (see Table 4). Indeed, if testing on many samples is required (e.g., in developing vaccines for viruses, ten thousand samples are often required), there is a need to create many (over 100) samples, which is not possible with the current approaches. Therefore, one technology gap can be concluded, namely:

○ ***Technology gap 1***: there is no technology available to create many (over 100) CFZs consistently.

### 3.2. Geometry of CFZs (R2)

Recent studies of wound healing have found that the chemical conditions (e.g., calcium concentration), coupled with the structural properties of wound area, can induce biological activities, which may affect healing [101]. Their study suggested that the structure of the environment of the cells may affect the healing process. However, the underlying principle of why and how the shape of CFZs matters with cell migration is not fully understood. As such, the following knowledge gap can be concluded, namely:

○ ***Knowledge gap 1***: there is no knowledge available to explain how and why different shapes of CFZs along with a chemical condition (e.g., calcium concentration) may affect the cell migration behavior in wound healing.

Further, Knowledge gap 1 leads to a new requirement on the CMA, i.e., creation of different geometric shapes of CFZs in CMAs. It is worth mentioning that the geometry of CFZs in the existing CMA is a two-dimensional area, which does not represent the complexity of the real situation of the wound, which says that the depth of the wound matters in vivo. In a pilot study by [32], a robotic device was developed to create CFZs at different geometric shapes including line, crosshead, rectangle, and triangle shapes (see Figure 2 in Section 2.1.1). However, this device has limitations. First, the device can only make one CFZ. Second, the device has a limitation in the shear stress condition. In the study of [32], the mechanical force on the cells was created by magnetic force. The magnetic force can be decomposed into the vertical force to apply pressure on cells, and horizontal force to drag the device moving (see Figure 2). In this case, the device has three functions: (1) to create CFZs by applying the pressure on cells, (2) to drag the device moving to create different shapes, and (3) to detach cells from the substrate. On one hand, creating CFZs needs a high pressure (about 5 bar) to the wound cells [30]. On the other hand, a high magnetic force for driving the device creates an “inertia” effect, resulting in disturbance in the fluid, which causes the device to move slowly (2 mm/s) in the steady flow pattern. Therefore, the disturbance can cause a serious problem in both force generation and measurement at the shear stress condition, which is focused on in the study of [30].

Different shapes of CFZs may raise another challenge in terms of measuring the “additional” stress created due to different shapes of CFZs. This is because such a stress is very small, thereby demanding high-resolution (certainly nano-scale level) microscopy. The foregoing discussion thus leads to the following technological gap:

○ ***Technology gap 2****:* there is no technology available to make different shapes of CFZs for many CFZs in CMAs.

### 3.3. Uniformity and Adjustability of Fluid Shear Stresses in Cells (R3 and R4)

The existing studies focus on the control of fluid-induced pressure to manipulate shear stress in cells by using pumps or valves [50]. As discussed in Section 2.3.2, the pathway for force transfer from the external pressure to the single cell is composed of various protein and signaling molecules at the scale of 1 to 140 nm (see Figure 4), while the microfluidic device is at the scale of about 5 μm. Therefore, the current state-of-the-art microfluidic device has two drawbacks: (a) difficulty to generate force and pressure on cells in high resolution (or nanoscale), and (b) difficulty to measure stress and strain in cells in high resolution (or nanoscale).

Regarding (a), the state-of-the-art devices may refer to the microfluidic devices developed by Lin et al. [33] and Vivas et al. [52]. In the first device, the pump, which generates the pressure on cells, is from an external pump, which is connected to the device. This connection is inherently bulky, becoming a limiting factor to the level of resolution, namely the microscale only. In the second device, the pump, as well as the valve, is within the device, specifically with the embedded PDMS membranes in the device, functioning as a kind of integrated microfluidic circuit (IMC) [102]. Such an integrated device indeed has the potential of achieving a nano-resolution pressure transfer to cells (i.e., creating nano-resolution stress in cells). However, the issue is sustainability due to the degradation of the PDMS membrane, which has a 14-day duration according to the report by Vivas et al. [52].

Regarding (b), two state-of-the-art methods are available: probe-based and interaction-based methods. The probe-based method, such as atomic force microscope (AFM), is used to measure the interaction force between a probe tip and a cell surface [50]. This method can achieve the measurement resolution at the scale of 0.4–1 μm (sub-cellular level), but this method is highly intrusive to cells. The second method is used to measure the interaction force between cells and synthetic environments (i.e., substrate or matrix) [60]. The main challenge with this method is its robust accuracy, as relatively huge disturbances and noises can come from the environment. The above discussion may conclude the following technological gap, namely:

○ ***Technology gap 3***: there is no technology available to generate and measure local stress in many cells (around 1 ×$\;10^{6}$/mL in cell density) in nano-scale accuracy.

### 3.4. Multiple Conditions and Samples (R5)

The state-of-the-art CMA has paid attention to the number of biological and chemical conditions. Most CMAs require repeating experiments to obtain a few samples from each well, while other CMAs can produce multiple samples from the device rather than by repeating the experiment. As shown in Table 4, the device developed by Yue et al. with the commercial CMA (${\mathrm{Oris}}^{\mathrm{TM}}$ CMA) can produce 12 chemical conditions in total, with eight samples in each condition [28]. Lee et al. developed a device (see Figure 1c), which has one condition and produces 20 samples [27]. It is noted that the fixing number of 96 is empirical. Some applications may only need a few conditions, e.g., 10 conditions, and as such, the capability of 86 conditions is wasted with the device with a fixed number of conditions. Therefore, the CMA should be adjustable to a varying number of conditions depending on applications and should be able to produce a varying number of samples without the need for repeating the tests. The following technological gaps can be derived:

○ ***Technology gap 4***: the current CMA can provide the 96 conditions only, which may have far more conditions than that which an application needs, e.g., 10, thereby increasing extra costs to the application (due to unused capacity);
○ ***Technology gap 5***: there is no technology available to build a CMA that allows for multiple conditions and, in the meantime, produces multiple samples;
○ ***Technology gap 6***: there is no technology available to build a CMA that allows adjustable multiple conditions and, in the meantime, produces adjustable multiple samples.

## 4. Limitation of CMAs to Wound Healing Assays

The use of the CMA to study wound healing assays does not cover all the attributes that represent a wound-healing process in a specific context, e.g., drug development and testing [6]. In general, wound healing assays are complex physiological models [103]. The context of wound healing assays has the physiological conditions of tissues along with possible other health complications such as arterial disorders [104,105]. In contrast, the CMA can only represent an aggregated mechanical behavior (movement) of a mechanics-related wound (e.g., scratching of skins). The following are specific examples of the limitations with the CMA-based wound healing assay.

(1) The existing CMAs have too short operation durations as opposed to the duration of wound healing in practice, e.g., over 21 days for chronic wound healing [106] and 7–21 days for maintaining the physiological conditions [52].

(2) The existing CMAs cannot represent multi-cellular wound healing. It is noted that a wound may happen with cells of different types, e.g., immune cells, fibroblasts, endothelial cells, and keratinocytes [107]. To model situations of multi-cellular wound healing, the CMA needs to be built into a 3D device. There are some efforts in the literature on designing and building 3D CMAs, e.g., [108,109], without much success.

(3) The existing CMAs cannot represent multiple stimuli on cells and their responses, which is a basic approach to understanding wound healing. It is noted that wound healing involves several phases of cellular activities, such as cell growth, migration, and differentiation [2], and additionally, these activities are highly coupled. For example, during the migration of cells, cells may take nutrients for growth [6]. Therefore, the movement behavior in such a situation is influenced by two factors: healing and growth. Further, this means that the measured movement outcome does not reflect the healing only [17]. In their approach, cell growth is required to be inhibited to measure the migration. However, cell growth is a fundamental biological function in the process of healing.

## 5. Conclusions and Future Research Directions

This paper presented a critical review of the CMA along with its application to building the wound healing assay. This paper took a system’s view to the CMA. We took the wound healing assay to derive the requirements on CMAs. As such, the overall requirement for the CMA was derived, which was further decomposed into several sub-requirements in terms of processes taking on the CMA. Knowledge and technology gaps were derived and presented in Section 3 above. Future studies are thus proposed to close the gaps as well as to further advance the CMA technology, and they are presented in the following:

*(1)*  *Further improving consistency of creating many CFZs (Technology gap 1)*

The CMA in [31] created 400 samples based on the approach of creating CFZs simultaneously. This approach is very promising for creating many samples. However, the device has a limitation in consistency. Currently, there is no effective method to clean cell debris in the mechanical approach of depletion. The consistency is thus compromised due to “unclean” cell debris in many samples. Future work will be focused on improving consistency using the chemical approach after the mechanical depletion to build CFZs.

*(2)*  *Advancing our understanding of the interactions of different geometric shapes of CFZs along with chemical conditions and their effects on cell migration in wound healing (Knowledge gap 1)*

The current understanding in the literature is that chemical conditions with different shapes of wounds do not have a significant impact on cell migration in the process of healing [110]. A hypothesis of cell migration with responses to different geometric shapes of cell environments may be found from mechanical conditions.

*(3)*  *Development of devices meet the functional requirement of changing the different shapes of CFZs for many CFZs (Technology gap 2)*

As discussed in Section 3.2, the device developed by Yimaz et al. [32] creating different shapes of CFZs has many shortcomings due to the ad hoc design process. In the shear stress condition, the device could cause disturbances in both force generation and measurement. From a design point of view, the design of the device in [32] was a coupled design, which is not an acceptable design based on the design theory called axiomatic design theory (ADT) [19,111]. Further work is needed to apply a Systematic Design Procedure (SDP) [18,26].

*(4)*  *Optimization of the structural design of reducing variations (Technology gap 3)*

There are many sources of disturbances in the CMAs. In particular, the disturbances from many cells moving create variations. To address the issue of the variations, a well-known design method, called Taguchi’s robust design method [112], will be employed to identify the design parameters in structural design (also known as embodiment design of a device). With a given design of the structure of the channel, the purpose of the robust design is to make the device less sensitive to the variations. Future work will be focused on advanced modeling based on multiscale and multi-physics simulations enabling optimization of the structural design [113,114].

*(5)*  *Modularization of existing components in the CMA (Technology gap 4)*

The creation of the chemical conditions as well as samples takes geometric space. For example, the microfluidic device developed by Monfared et al. (2020) consists of five substrates, and each substrate (76 mm × 26 mm) has eight conditions and four samples at an area of about 1.8 mm^2^. One idea is to expand the footprint of CMAs. However, the microfluidic device has a restriction at micro-size scale (100 nm to 100 μm) for the accuracy of fluid control and cell manipulation [115]. Another idea is to use a so-called modular approach [116] to miniaturize the device system. Millet et al. used the modular approach to miniaturize a microfluidic device for the process of protein purification [117]. By reconfiguring the fluidic operation and biological chemical process along with the existing components, the miniatured device was able to detect a small volume of proteins. More previous studies in the miniaturization of microfluidic devices in medical application can be found in [118]. Future work will be focused on the application of the modular design approach for the miniaturization of the CMA to increase the number of conditions.

*(6)*  *Development of CMAs to adjust the number of conditions as well as the number of samples (Technology gap 5,6)*

Taking the modular approach requires standardizing the process along with devices [119]. In the CMA, the modular approach can standardize the process along with functional components (e.g., pumps, channels, etc.). Future work will use the system and design thinking to develop design guidelines for building a platform systematically to allow designers to customize the number of conditions as well as samples in the CMA.

*(7)*  *Combination of multiple conditions to create a more biomimetic environment*

Wound healing is a multivariate process involving biological responses under multiple stimuli. This calls for the development of devices to create more biomimetic in vivo conditions by combining them with different aspects of stimuli to the wound-healing process. Previous studies combining different biological, electrical field, and chemical conditions can be found in [120]. Several studies investigate the effects of different surface coatings on the wound-healing process [121,122]. However, this requires further investigation of the surface coatings in relation to the properties (e.g., biocompatibility) of PDMS-based CMAs.

*(8)*  *Extension of CMAs for other biomedical applications*

The CMA is a generic device while the wound healing assay is a specialized device to use the CMA to study the wound-healing process. Cell migration is a cell’s behavior that has potential for other applications, e.g., tissue regeneration [123], drug screening and testing [124], toxicity [125], and chemotaxis studies [126,127].

## Figures and Tables

**Table 1:** Classification of the existing devices creating CFZs (FR1) based on the principles.

Principle (Level 0)	Operation Methods	Number of Samples	Size of CFZs	Consistency	Pros	Cons	References
Mechanical (DP1-1)	Manual	1	800 μm width	Poor	Simplicity	Irregular size	[10]
	Manual	20	0.06 mm^2^	6%	Easy fabrication	Cell debris	[27]
	Manual	8	600 μm width	Poor	Availability	Lack of shear stress	[28]
	Auto	4	0.91 mm^2^	4%	Automation	Bubble formation	[29]
	Auto	40	1.5 mm^2^	5%	Controllable force	Bubble formation	[30]
	Auto	400	0.126 mm^2^	4%	Large number of samples	Few conditions	[31]
	Auto	1	500 μm width	2%	Different shapes of CFZs	Low efficiency	[32]
Chemical (DP1-2)	n/a	1	3–6 mm width	5%	Clean CFZs	Affecting cell dynamics	[33]
Electrical (DP1-3)	n/a	1	0.05 mm^2^	n/a	Easy to control the power	Generating heat	[34]
Thermal (DP1-4)	n/a	Few	5–20 mm^2^	Poor	Thermal wounding	Chemical reaction	[35]
						Stressed cells	
Optical (DP1-5)	n/a	96	2 mm^2^	Good	Large samples	Costly equipment	[36]

**Table 2:** Classification of the existing devices measurement of cell migration (FR2).

Method	Resolution	Pros	Cons	References
Label-based (DP2-1)	0.4–1 μm	Automated imaging Easy to track	Invasive Thin samples (~10 μm)	[45] [46]
Label-free (DP2-2)	Poor at single cell (~5 μm)	Samples depth up to 70 μm Non-invasive	Intensive for tracking Low resolution and labor	[47] [48]

**Table 3:** Classification of the devices for creating the mechanical conditions required (FR3).

Principle (Level 1)	Principle (Level 2)	Materials	Stress	Pros	Cons	References
Fluid (DP3a-1)	Electrical	PDMS	<25 dyn/cm^2^	Stable power	Non-uniform fluid distribution	[49,50,51,52,53,54,55,56]
	Electromagnetic	PDMS	20~60 kpa	Easy to control	Temperature rise at high electric current (~300 mA)	[57]
	Pneumatic	PDMS	102.05 μL/min	Biocompatible	Formation of bubble	[58]
Cells (DP3a-2)	Mechanical	Hydrogels	2–10 kPa	Biomimetic	Limited force range (~13 mN)	[59]
	Chemical	Growth factors	0.1–50 kPa	Chemical conditions	Poor cell dynamics	[60]
Environment (DP3a-3)	Physical	Electrodes magnetics optics acoustics	2–10 kPa	Sufficient power	External energy Lack of modeling in first principle	[59,61,62,63,64,65]
	Chemical	PH ions oxygen	0.1–40 kPa	Easy targeting	Longer reaction time Confounding factors with other chemicals	[66,67,68,69,70]
Probe (DP3b-1)	n/a	Probe	5 pN to 10 nN	High resolution (0.2 μm)	Few cells Light contamination	[71]
Interaction (DP3b-2)	n/a	Fluorescence	2–120 nN, 0.05–0.6 kPa	Large number of cells (>1000)	Difficult to measure on single cell	[60]

**Table 4:** Overview of the state-of-the-art CMAs.

Requirements						References
Consistency * (R1)	Geometry (R2)	Shear Stress (R3)	Uniformity (R4)	Number (R5)		
				**Samples**	**Conditions**	
Poor	Line	n/a	n/a	1	1	[10]
6%	Line	n/a	n/a	8	12	[28]
Poor	Square	n/a	n/a	20	1	[27]
n/a	n/a	0.01 dyn/cm^2^	90%	n/a	1	[56]
4%	Circle	3 μL/min	n/a	4	4	[29]
n/a	Line	1~18.3 dyn/cm^2^	n/a	3	3	[33]
2~5%	Circle	n/a	n/a	4	8	[30]
4%	Circle	1–7 dyn/cm^2^	66%	400	1	[31]
2%	Multiple	20 μL/min	n/a	1	1	[32]

* The consistency is defined as relative standard deviation (RSD) in Section 2.1.1. It is noted that Table 4 overlaps with Table 1 and Table 3 in terms of R1, R3, and R4.

## Data Availability

Data will be available based on request.

## Supplementary Materials

The following supporting information can be downloaded at: https://www.mdpi.com/article/10.3390/mi15060720/s1, Figure S1: The general design process model for device development; Figure S2: The definition of FR along with its decomposition; Figure S3: The example of the design result, i.e., the FR-DP relation.
