# Supplementary material for: Cell Migration Assays and Their Application to Wound Healing Assays—A Critical Review
Source: Micromachines (Basel). 2024 May 29;15(6):720. doi: 10.3390/mi15060720 (PMC11205366; doi:10.3390/mi15060720)
Supplement: Supplementary file 1 [file micromachines-15-00720-s001.zip › micromachines-2873497-supplementary.pdf]

## Supplementary Material

### Design and design process:

In design literature (Suh, 2010), the design process involves two domains: The Function Requirement (**FR**) domain and Design Parameter (**DP**) domain. A design represents a mapping (**Φ**) from FR to DP. A design process is to build this mapping and is further divided into a general design process and a specific design process. A well-known general design process divides a whole design process into four design phases (Pahl et al., 2007; Dai, 2019; Zhang and Wang, 2016; Zhang et al., 2019; Zhao et al., 2022):

- Phase 1: Development of the technical specification of requirements from the customer's voice of requirements.
- Phase 2: Concept design.
- Phase 3: Embodiment design.
- Phase 4: Detail design.

The above design phases are viewed as the design phase dimension (Figure S1). An orthogonal dimension is the decomposition of requirements into sub-requirements. Specifically, for each sub-requirement, concept design, embodiment design and detail make sense to it. A specific design process refers to the application of the knowledge only relevant to specific devices or systems, e.g., bike or cell migration assay (CMA) etc., under design. A specific design process substantiates each phase of the general design process as well as the decomposition of requirements. This means that designs take place at each design phase as well as the requirement decomposition is performed all with the specific design knowledge to a particular device.

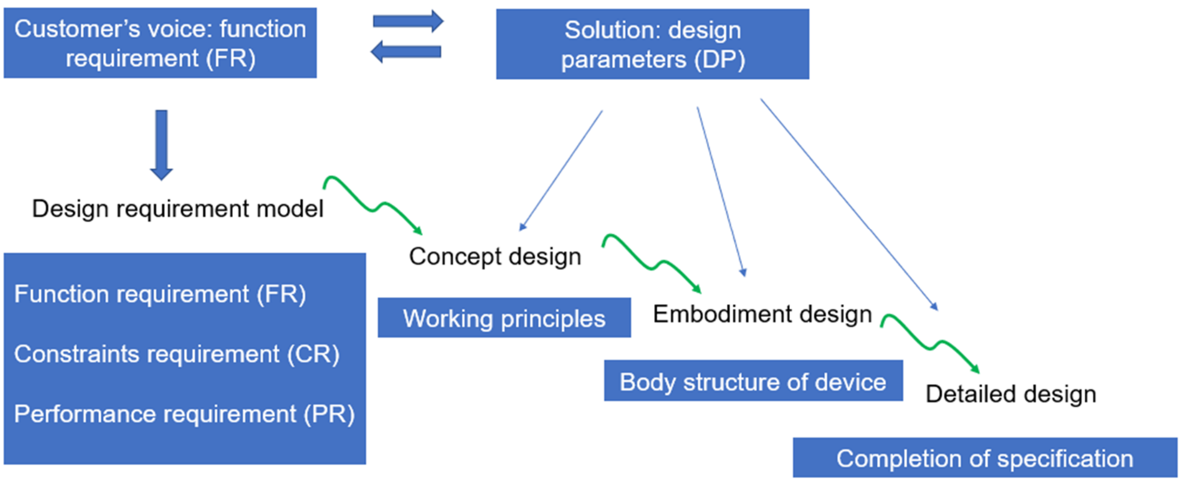


Figure S1. The general design process model for device development.

**Design requirement:**

A design requirement consists of function reequipment (**FR**), performance requirement (**PR**), and constraint requirement (**CR**). Both PR and CR are on the top of FR. Figure S2 illustrates the notation of FR along with the dimension of reequipment (function) decomposition. FR1 and FR2 stand for the 1$^{st}$ function at Level 1 and the 2$^{nd}$ function at Level 1. The number of dots[1] '.' represents the number of levels minus one. It is further noted that the dot '.' for Level 1 is omitted. For example, FR2 means that the second function at Level 1, FR1.2.1 stands for the 1$^{st}$ function at Level 3 along with the hierarchy of decomposition (i.e., the parent function of FR1.2.1 is FR1.2, which represents the second function at Level 2, and the parent function of FR1.2 is FR1, which represents the first function at Level 1).

[1] It is noted that in this study, the dot '.' and the dash '-' are used interchangeably.

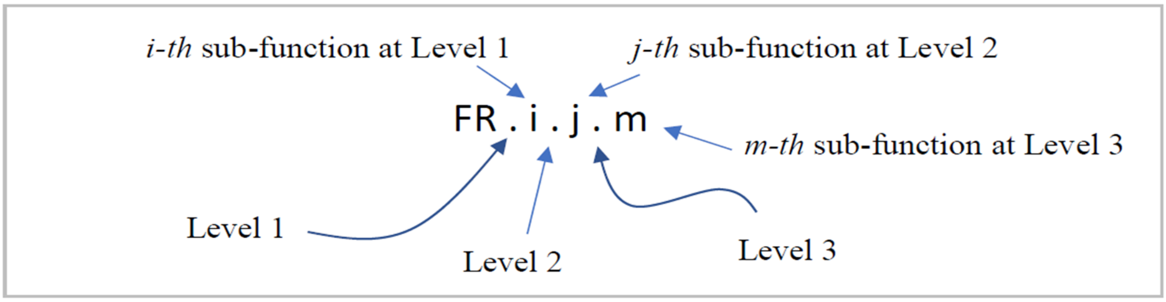


Figure S2. The definition of FR along with its decomposition.

**Design description and design result:**

The description of the result of design or design description is denoted as Design Parameter (DP) for brevity. The notation of DP follows that of FR. For instance, DP1.2.1 means the description of the result of design to meet the requirement, FR1.2.1. The DP is applicable to all the design phases (concept design, embodiment design, and detail design). Figure S3 shows the example of the design result, i.e., the FR-DP relation. To DP, there may be several alternatives, all of which can meet the FR, and this semantics can be represented by DP.i.j[a], where 'a' denotes an alternative design. For instance, DP1.2a and DP1.2b stand for two design alternatives to FR1.2.

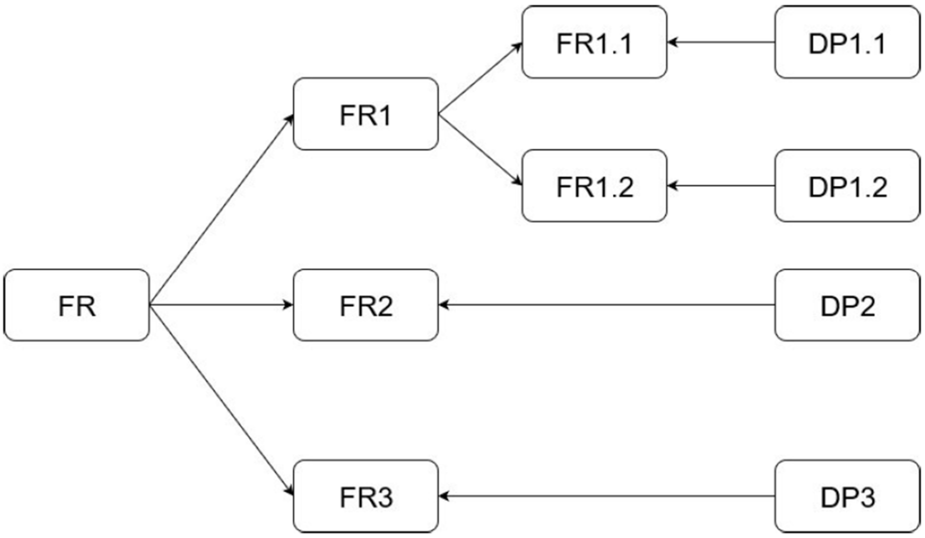


Figure S3. The example of the design result, i.e., the FR-DP relation.

**Design thinking and its application in reviewing literature:**

Elsewhere, we propose the so-called design thinking (DT). DT is not about design, but it is about solving a problem by viewing the problem as a design problem. As such, the general theory and methodology for design can be applied, e.g., the design phase theory mentioned before. The DT is applied to developing an understanding of the literature on a subject of interest, e.g., CMA, resulting in an approach to developing a literature review paper, and this approach was first attempted in our previous paper, see (Tony et al., 2023), coined as DTRL (DT methodology for Reviewing Literature).

We consider that a literature review paper ought to be valuable if it can achieve two goals of information provision: (1) to identify knowledge and technology gaps and (2) to propose further directions of research to close the gaps as well as to go beyond (i.e., a new question, new

hypothesis). It is further noted that we define the science as knowledge to discover or hypothesize or to account for phenomena or to explain causes for effects, the methodology and technology as a tool to make and operate devices that serve for humans, and the device as the result of applying the methodology and technology. Therefore, the methodology for developing a literature review paper on the subject of science, technology, and device with the DTRL may be different.

To write a literature review paper on the subject of a device with the DTRL to meet the goals of a review paper (see the previous discussion in this document), our methodology consists of the following steps.

- Step 1: Understand the requirement for the device (CMA in this case) to be reviewed. It is noted that the requirement may be a hierarchy of sub-requirements.
- Step 2: Classify (including categorize) the literature in terms of design of the device (CMA in this case). This means that the classification will be based on the DP, especially at the concept design phase, where the working principle of a device is concerned.
- Step 3: Analyse the literature, various designs of CMA from the literature in this case, against the requirement, established in Step 1. The result of this step prepares for deriving knowledge and technology gaps.

There are a couple of benefits with Step 2. First, for each work related to design of CMAs, with the classification we can know which category the work falls into, and this helps to understand the effectiveness of the working principles or concepts of the device under design or concern. Second, it enables to explain the achievement (i.e. the actual performance and functionality under the

constraint requirement) of a design with respect to the requirement from its working principle, which means a deep understanding of knowledge and technology gaps if any.

## References for supplementary material:


[S1] Pahl, G.; Beitz, W.; Feldhusen, J.; Grote, K.H. Engineering design-a systematic approach, 3rd ed.; Springer, **2007**; 159.

[S2] Suh, N.P. The principles of design, New York: Oxford University Press, 1990.

[S3] Tony, A.; Badea, I.; Yang, C.; Liu, Y.Y.; Wells, G.; Wang, K.M.; Yin, R.X.; Zhang, H.B.; Zhang, W.J. The additive manufacturing approach to Polydimethylsiloxane (PDMS) microfluidic devices: review and future and directions. *Polymers* **2023**, 15 (8), 1926

[S4] Zhao, Y.; Zhu, D.; Zhou, F.; Song, J.; Zhang, W. J. An axiomatic design theory for design of apparel products. *J. Eng. Fibers Fabr.* **2022**, 17, doi:10.1177/15589250221134350.

[S5] Zhang, W.J.; Wang, J.W. Design theory and methodology for enterprise systems. *Enterp. Inf. Syst.* **2016**, 10 (3), 245-248.

[S6] Zhang, W.J.; Wang, J.W.; Lin, Y. Integrated design and operation management for enterprise systems. *Enterp. Inf. Syst.* **2019**, 13 (4), 424-429.
